# Investigating the Role of Translationally Control Tumor Protein in Growth, Development and Differentiation of *Dictyostelium discoideum*

**DOI:** 10.3389/fcell.2020.00742

**Published:** 2020-08-07

**Authors:** Rakesh Kumar, Ranjana Maurya, Shweta Saran

**Affiliations:** Cell and Developmental Biology Lab, School of Life Sciences, Jawaharlal Nehru University, New Delhi, India

**Keywords:** *Dictyostelium*, development, growth, RT-PCR, TCTP, chimera

## Abstract

Translationally controlled tumor protein (TCTP) is a multifunctional protein implicated in various types of cellular processes involving growth and development of an organism. Here, we identified *tctp* gene in *Dictyostelium discoideum* and unraveled its function. The sequence analysis of *D. discoideum* TCTP (*Dd*TCTP) showed its conservation among eukaryotes. Transcript of *Dd*TCTP was highly expressed at the initial time points of development and protein is localized both in the cytoplasm and nucleus. Disruption of *tctp* was achieved by BSR cassette using double homologous recombination method. Abrogation of *tctp* resulted in reduced cell proliferation but increased cell size. Additionally, development was delayed by 4 h wherein small-sized aggregates and fruiting bodies were produced by *tctp*^–^ cells while larger aggregates and fruiting bodies were produced by *tctp*^*OE*^ cells concordant with the fact that TCTP regulates prestalk/prespore ratio and cell-type differentiation. *tctp*^–^ cells produced round spores with reduced viability and stalk cells are arranged in septate pattern as compared to polyhedral manner of wild type. Abrogation of *tctp* resulted in aberrant localization of cell type specific markers and show low proclivity toward prespore/spore region, in presence of wild type cells.

## Introduction

Translationally controlled tumor protein (TCTP; also known as P21, P23, and fortilin) is implicated in various biological activities such as cell growth, cytoskeleton regulation (Gachet et al., 1999; Burgess et al., 2008; Tsarova et al., 2010), cell cycle (Brioudes et al., 2010), protein synthesis (Cans et al., 2003), apoptosis (Li et al., 2001; Liu et al., 2005; Yang et al., 2005), development (Chen et al., 2007; Hsu et al., 2007), and cancer (Tuynder et al., 2002, 2004; Rho et al., 2011). TCTP has a critical role in tumorigenesis, which is now recognized as a therapeutic target for various cancers such as prostate, lungs and breast cancers (Acunzo et al., 2014). TCTP is a ubiquitously expressed highly conserved protein present in eukaryotic system from plant to yeast and protozoans to all invertebrates and vertebrates and involved in various kinds of biotic and abiotic stresses (Rupec et al., 1998; Sturzenbaum et al., 1998; Cao et al., 2010). It is crucial for development of an organism as the knockout in mice is lethal where the fetus dies in the uterus due to inhibition of cell proliferation and increased cellular apoptosis (Chen et al., 2007; Susini et al., 2008). The embryo suffers reduction in cell number and increased apoptosis at embryonic stage 5.5 and dies around stage E9.5–10.5 (Chen et al., 2007). TCTP mutant mice show defect in neuronal and glial differentiation during CNS development (Chen et al., 2020). Also, knockdown of TCTP in *Drosophila* results in reduced cell number and organ size causing the larvae to die by first-instar stage (Hsu et al., 2007). High abundance of TCTP transcript is found in retinal ganglion cell and adult axons indicate TCTP regulates the growth of retinal ganglion cell axons (Roque and Holt, 2018). It is associated with the survival machinery of axons and has role in the development of vertebrate neural circuit (Roque et al., 2016). In *Drosophila*, RNA interference of TCTP causes a reduction in size of eye and wing which are due to decrease of both cell number and size. It also helps in the development of brain, wing and endocycling cells of salivary glands of *Drosophila* by maintaining the genome stability of mitotic cells (Hong and Choi, 2013). In plant or *Arabidopsis*, downregulation of TCTP results smaller leaf and flower size, reduced root growth, and leads to delayed development (Berkowitz et al., 2008). TCTP also controls the cytoskeleton, such as microfilament and microtubule by interacting with actin and tubulin proteins (Gachet et al., 1999). Hence, TCTP plays a critical role in cell proliferation in a cell or tissue-type specific manner.

*Dictyostelium discoideum* encompasses the unicellular and multicellular life forms that makes it tractable for the study of genetic changes occurring at the intersection of uni- and multicellular life (Eichinger et al., 2005). It has been used for the study of chemotaxis, cell biology, and developmental biology (Firtel and Chung, 2000; Cardelli, 2001). In metazoans, multicellularity results by the repeated cell division of the “zygote” but in case of *Dictyostelium* multicellularity arises due to the aggregation of spatially segregated cells. *Dictyostelium* feeds on bacteria and divides mitotically to remain as unicellular organism but enters into multicellularity at the onset of starvation (Loomis, 2014). Following starvation, some of the cells start secreting cAMP and in response to this signal other cells start moving toward cAMP gradient (Saran et al., 2002). These cells communicate with each other and form aggregation territories (mounds) comprising of approximately 10^5^ amoebae which undergo various morphogenetic movements to form slugs comprising anterior 1/_4_ region of prestalk cells and the remaining posterior region of prespore cells and then fruiting bodies that made up of dead vacuolated stalk cells and viable spores (Raper, 1935; Wittingham and Raper, 1960; Williams et al., 1989).

In this paper, we have identified and characterized *Dictyostelium* TCTP (*Dd*TCTP) that show conservation to human and yeast TCTP. Disruption of *Ddtctp* was achieved using Blasticidin (BSR) cassette to unravel its function in growth and development of this organism. The *tctp*^*OE*^ (overexpressor) and *tctp*^–^ (knockout) cells showed reduced cell proliferation. Disruption of *tctp* exhibited increased cells mass, cell size and reduced pinocytosis. Both, *tctp*^*OE*^ and *tctp*^–^ cells displayed delay in development resulting in fruiting body formation at 28 h, respectively. Additionally, *tctp*^*OE*^ cells formed large-sized aggregates and fruiting bodies with a larger stalk and a small sorus. On the contrary, *tctp*^–^ cells formed small-sized aggregates and fruiting bodies. *tctp*^–^ fruiting bodies formed round spores in contrast to elliptical spores of wild type and exhibited only ∼25% viability. In chimeras of Ax2 and *tctp*^–^ cells, mutant cells showed more propensity toward prestalk/stalk region and its derived structures and contributed less in chimeric spore formation.

## Materials and Methods

### Cell Growth, Development and Transformation

*Dictyostelium* (Ax2) cells were grown axenically and development was performed as described (Gosain et al., 2012). Log phase cultures were inoculated into fresh media at a density of 5 × 10^5^ cells/mL and cell proliferation was monitored over 5 days using hemocytometer. For development, log phase cultures were harvested, washed and developed at a density of 5 × 10^7^ cells/mL, followed by synchronization at 4°C for 4–6 h and further incubated at 22°C to proceed in development. The timings and pattern of development was noted and photographed under Nikon SMZ-1500 stereomicroscope. Analyses were performed using NIS-ELEMENTS AR v. 4.0.

### Preparation of *tctp* Overexpressing (*tctp*^*OE*^) and *tctp* Knockout (*tctp*^–^) Strains

*Tctp* overexpressing (*tctp*^*OE*^) strain was made as described earlier (Kumar et al., 2017). The knockout of *tctp* gene was created by disrupting the *tctp* gene with BSR cassette, followed by homologous recombination in the genome of *D. discoideum.* The 5’ and 3’ homology regions were PCR amplified using gene-specific primer pairs (Supplementary Tables S1, S2) and cloned at the flanking sides of BSR cassette, followed by linearization of the construct (Supplementary Figure S1). The linearized product (5’ homology region + BSR cassette + 3’ homology region) was transformed into Ax2 cells by electroporation and transformants were selected at 10 μg/mL Blasticidin-S (Invitrogen).

### RNA Detection by RT-PCR Analysis

RNA isolation and cDNA synthesis were performed as described (Gosain et al., 2012). Briefly, *Dictyostelium* cells were developed and samples were collected at various time points, washed and lysed in TRI reagent (Sigma-Aldrich, United States). cDNA was synthesized and PCR amplification was performed. The gene-specific primer pairs used for RT-PCR reactions were mentioned in Supplementary Table S2.

### Pinocytosis Assay

Pinocytosis assay was performed as described (Maurya et al., 2020). Briefly, log phase cultures of Ax2 and *tctp*^–^ cells were harvested, re-suspended in fresh media at a density of 3 × 10^6^ cells/mL and kept under shaken conditions for 20 min. Furthermore, FITC dextran (2 mg/mL; Mr = 70 kDa; Sigma-Aldrich) was added and kept in dark under shaken conditions. 1 mL cell suspension was taken at different time points, washed with 1xKK_2_ + 5 mM sodium azide, and further by 1xKK_2_. Cells were lysed and fluorescence was measured at excitation 470 nm and emission at 520 nm. Samples were normalized with protein content to exclude any difference due to cell size.

### Cell Dry Weight Analysis

Cell dry weight measurement was done according to Maurya et al. (2017). Log phase cultures were harvested, washed and pellet having 5 × 10^7^ cells were vacuum dried at 55°C for 1 h and cell weight was scored by subtracting empty tube weight.

### Cell Size Analysis by Flow Cytometer

Cell size analyses were performed as described (Maurya et al., 2017). Briefly, approximately 1 × 10^7^ cells were harvested, washed with 1xKK_2_ buffer, resuspended in 1.5 mL buffer (0.9% NaCl, 2% sucrose, 5 mM EDTA in KK_2_ buffer) followed by fixation. Just prior to analysis 1 × 10^6^ cells were washed, treated with RNaseA and incubated at 37°C for 30 min followed by addition of propidium iodide (Sigma-Aldrich, United States) and incubated at RT. Sample was loaded on BD FACS Calibur flow cytometer and cell size analysis was performed using Cell Quest software.

### Spore Viability Assays

Spore viability assay was performed according to the procedure as described (Myre et al., 2011). Approximately, 1 × 10^8^ cells were harvested, washed and developed on 1.5% non-nutrient agar. Spores from mature fruiting bodies were collected, washed and aliquots of 100 spores were mixed with the suspension of *Klebsiella aerogenes* (bacteria) and allowed to grow for 5 days under moist conditions.

### Calcofluor Staining of Stalk and Spores of Ax2 and *tctp*^–^ Fruiting Bodies

Cells were developed at a density of 5 × 10^7^ cells/mL on nitrocellulose membrane, already placed on agar till the formation of fruiting bodies, followed by treatment with calcofluor white (Sigma) at 0.01% wt/vol for 10 min. The staining solution was removed and images were captured using fluorescence microscope (Nikon Eclipse TiE).

### Development of Chimeras

RFP marked Ax2 and GFP marked *tctp*^–^ cells were mixed in various proportions (10–75%) and developed as described above. The development of chimeras was monitored under fluorescence microscope (Nikon SMZ-1500) and photographed.

For scoring contribution of Ax2 and *tctp*^–^ cells in chimeric spore formation, individual spore-heads were picked on a glass-slide and photographed, both under brightfield and fluorescence using Nikon Eclipse 80i microscope. Red and green fluorescent spores were counted and graph was plotted. A minimum of 10–15 fruiting bodies developed from each ratio mixture per individual experiment were counted.

### β- Galactosidase Staining

β-galactosidase staining was performed according to the method described by Escalante and Sastre (2006) with minor modifications (Mishra et al., 2017). Log phase cells were developed at a cell density of 5 × 10^7^ cells/mL on dialysis membrane placed on non-nutrient agar plates. Various developmental structures were collected, fixed with methanol for 30 min followed by washing and permeabilization with NP40 (0.1% in Z buffer) for 1 h at room temperature. Further, structures were washed 2–3 times and staining with X-gal (5-bromo-4-chloro-3-indolyl-β-D-galactopyranoside) was performed. The reaction was stopped when evident staining appeared by washing with Z buffer. Images were captured under the Nikon AZ100 microscope.

### Image Processing and Statistical Analyses

Image processing and mound size analyses were performed using NIS ELEMENTS ARv.4.0. The statistical analyses were performed with mean, standard deviation, and standard error and values were plotted in graph using GraphPad Prism (GraphPad Software, CA, United States, www.graphpad.com). Student *t*-test was used where *p*-values; ^∗∗∗^*p* < 0.001, ^∗∗^*p* < 0.01 and ^∗^*p* < 0.05 were established as significant.

## Results

### Identification and Characterization of *Dd*TCTP

**FIGURE 1:**
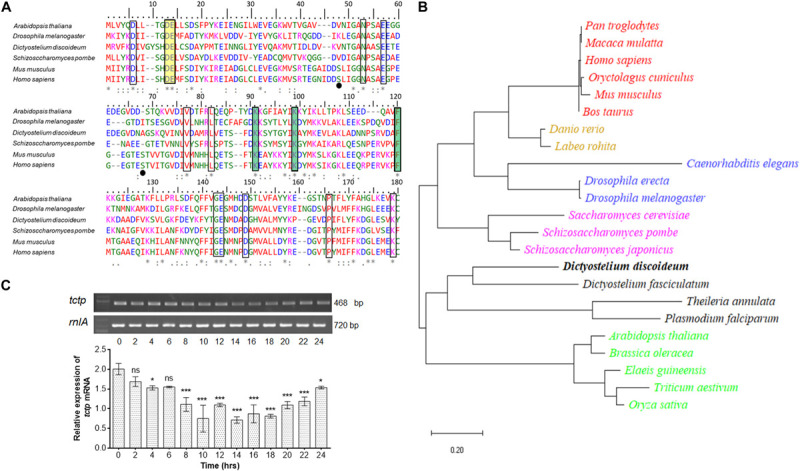
Identification and characterization of *Dd*TCTP. **(A)** Protein sequence alignment of *Dd*TCTP and other species. Sequences used in this study are: *Dictyostelium discoideum* (DDB0305046), *Arabidopsis thaliana* (Uniprot id: P31265), *Drosophila melanogaster* (Uniprot id: Q9VGS2), *Schizosaccharomyces pombe* (Uniprot id: Q10344), *Mus musculus* (Uniprot id: P63028), and *Homo sapiens* (Uniprot id: P13693). Identical [*], 90% conserved [:], and 50% conserved [.] amino acid residues are indicated in multiple sequence alignment. Residues highlighted (rectangular boxes) in different color involve in different functions which are conserved from lower to higher organisms. Residues crucial for Rheb interaction are shown in yellow color, while residues involved in microtubules and Ca^2+^ are highlighted in green color. Serine residues (Ser46 and Ser64) in mouse and human are known to be phosphorylated by the Plk (Polo like kinase) marked in black dots, are only conserved in mammalians. **(B)** Phylogenetic analysis of various TCTPs from protozoa and other species. Evolutionary distances between sequences are indicated as given scale in bottom. **(C)** Semi-quantitative RT-PCR of *tctp* transcript at different developmental time points. The values represent mean ± standard deviation; *n* = 3; ***p < 0.001, *p < 0.05.

To investigate the similarity between *Dd*TCTP and TCTPs from other species, we performed multiple sequence alignment of protein sequences through MUSCLE program (Edgar, 2004). The protein sequence of *Dd*TCTP is ∼171 amino acids (source from dictybase server; Fey et al., 2009). Amino acid sequence alignment of TCTP from social amoeba (*D. discoideum*) and other species (including *Arabidopsis thaliana, Drosophila melanogaster, Schizosaccharomyces pombe, Mus musculus*, and *Homo sapiens*) was performed (Figure 1A). We aligned the TCTP protein sequences from seven species that cover lower to higher animals, plant and fungi. Conserved residues showed identity between social amoeba, plants, fungi and lower as well as higher animals. A constructed phylogenetic tree suggested that *Dd*TCTP was closely related to the other members of dictyostelids and protozoa such as *Theileria annulata, Dictyostelium fasciculatum*, and *Plasmodium falciparum* with high bootstrap values (Figure 1B). *Dd*TCTP was also closer to fungi (*Saccharomyces cerevisiae*, *Schizosaccharomyces japonica*s, and *Schizosacchromyces pombe*). The homology between *Dd*TCTP and *S. pombe* was 46% while with human it showed 42%.

The expression level of *tctp* mRNA during growth and development of *D. discoideum* was checked using semi-quantitative RT-PCR (Figure 1C). *tctp* mRNA was expressed throughout development, predominantly expressed at initial time points (0–6 h) then showed reduced expression between 8 and 20 h followed by increased expression till 24 h.

### Generation of *tctp* Knockout in *Dictyostelium discoideum*

**FIGURE 2:**
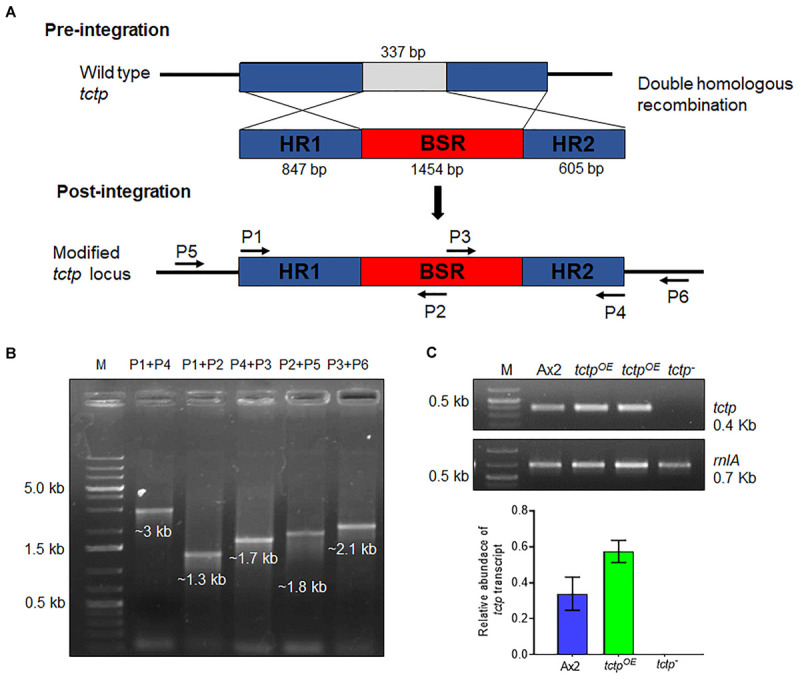
Generation of *tctp* deletion mutant. **(A)** Schematic representation of *tctp* disruption using BSR cassette (blasticidin resistance cassette) employing double homologous recombination method. Linearized product (5’ homology region + BSR + 3’ homology region) was transformed into Ax2 cells and transformants were selected at 10 μg/mL blasticidin. **(B)** Confirmation of *tctp*^–^ clone by PCR using primer pairs for positional integration. **(C)** Semi-quantitative RT-PCR to check *tctp* mRNA in Ax2, *tctp*^*OE*^, and *tctp*^–^ cells.

To explore the functions of TCTP, its deletion mutant was generated by disrupting the *tctp* gene with BSR cassette through homologous recombination method in the genome of *D. discoideum* (Figure 2A and Supplementary Figure S1). Approximately, 400 independent clones were screened for positional integration, confirmed by PCR and one out of the two positive clones was used for further experiments (Figures 2B,C).

### *Dictyostelium* TCTP Reduces Cell Proliferation

**FIGURE 3:**
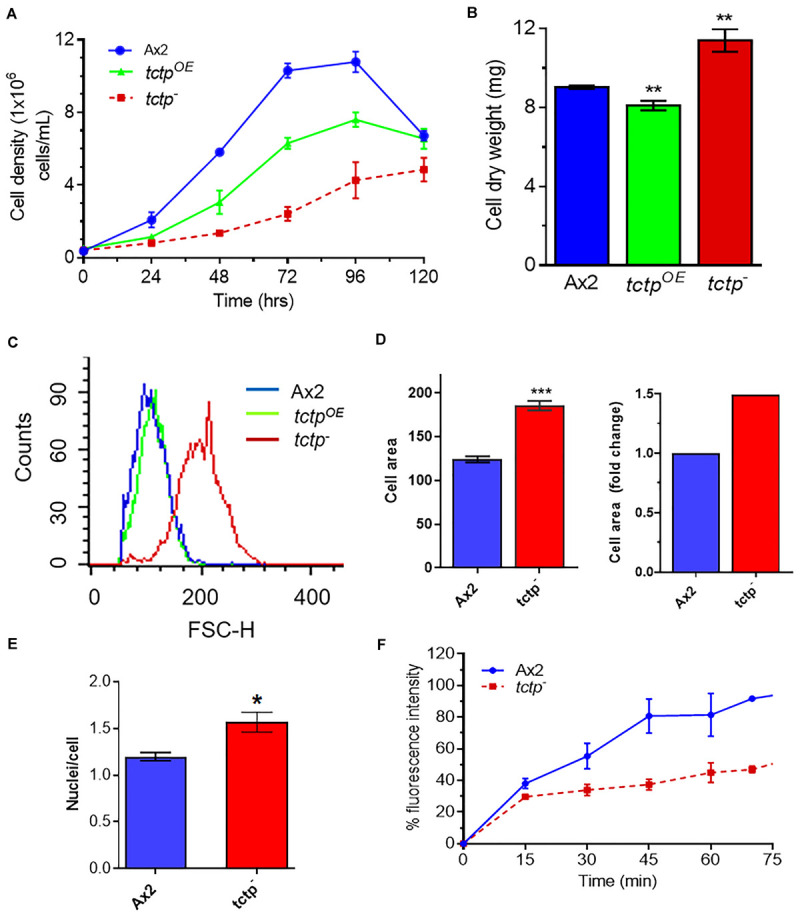
Abrogation of TCTP rendered enhanced cell growth but reduced cell proliferation. **(A)** Cell proliferation rate of Ax2, *tctp*^*OE*^, and *tctp*^–^ cells was measured every 12 h. (Seeding density- 5 × 10^5^ cells mL^–1^). **(B)** Cell dry weight analyses of Ax2, *tctp*^*OE*^, and *tctp*^–^ cells. Cell dry weight was computed in milligram. **(C)** Relative cell size analyses of Ax2, *tctp*^*OE*^, and *tctp*^–^ cells were carried out by FACS. **(D)** Measurement of cell size of Ax2 and *tctp*^–^ **(E)** Quantification of nuclei per cell of Ax2 and *tctp*^–^ cells. **(F)** Pinocytosis measurement by FITC-dextran uptake assay in Ax2 and *tctp*^–^ cells. (The values represent mean ± standard error mean; *n* = 3; ***p < 0.001, **p < 0.01, *p < 0.05; Student’s *t*-test; and Scale bar- 10 μm).

For measuring cell proliferation, log phase cultures were identically diluted into fresh HL5 media and observed over 5 days (Figure 3A). Wild type and *tctp*^*OE*^ cells divides at every 12.3 ± 0.2 and 18 ± 0.6 h and attained stationary phase at ∼10.8 × 10^6^ cells/mL and ∼7.2 × 10^6^ cell/mL, respectively, while cell proliferation of *tctp*^–^ cells was very slow with a longer lag phase and reached the stationary phase and at a much lower density of 4.7 × 10^6^ cell/mL (Figure 3A). The doubling time for *tctp*^–^ cells was 26 ± 2.5 h. Growth of *Dictyostelium* was accomplished with either increase in cell proliferation or increase in size of the cells. Cell growth and proliferation are regulated independently in the organism. Cell proliferation rate is increase in number while cell growth is increase in size of cell due to the increased molecular mass in the cell. To determine the role of *Dd*TCTP in regulating growth, we estimated the cell mass and observed that Ax2, *tctp*^*OE*^, and *tctp*^–^ cells have an average cell mass of 9 ± 0.08, 8.1 ± 0.2, and 11.4 ± 0.5 mg, respectively (Figure 3B). Increased cell mass of *tctp*^–^ cells was attributed to the enhanced cell size revealed by FACS analysis (Figure 3C). The size of the *tctp*^–^ cells was also observed by microscopy and found significantly more than wild type cells (Figure 3D and Supplementary Figure S2). Furthermore, the reason behind increased cell size was checked via nuclear staining using DAPI and found that there was increase in number of nuclei per cell in case of *tctp*^–^ cells as compared to wild type cells (Figure 3E). Together, we conclude that *tctp*^–^ cells display reduced proliferation but enhanced cell growth due to increased number of nuclei. Next, we wanted to check the effect of TCTP knockout on pinocytosis (rate of liquid uptake; Figure 3F). Pinocytosis also known as macropinocytosis is the uptake of liquid media by axenic strain (Ax2) of *Dictyostelium* cells grown in laboratory. Pinocytosis is a crucial process in the growth of *Dictyostelium* Ax2 cells, as it is the only mode of taking the nutrients (Bloomfield and Kay, 2016). Vegetative stage of Ax2 cells grow and divide by actively pinocytosing the external nutrients. It can be measured through the rate of uptake of fluid phase marker, fluorescent dextran (Maurya et al., 2020). The pinocytosis rate of *tctp*^–^ cells was significantly reduced at all time points as measured by decrease in intracellular FITC content in the *tctp*^–^ cells (Figure 3D). Therefore, decreased proliferation could be attributed to defect in nutrient uptake as well.

### Deletion of *tctp* Cause Developmental Delay and Aorist Fruiting Bodies Formation

**FIGURE 4:**
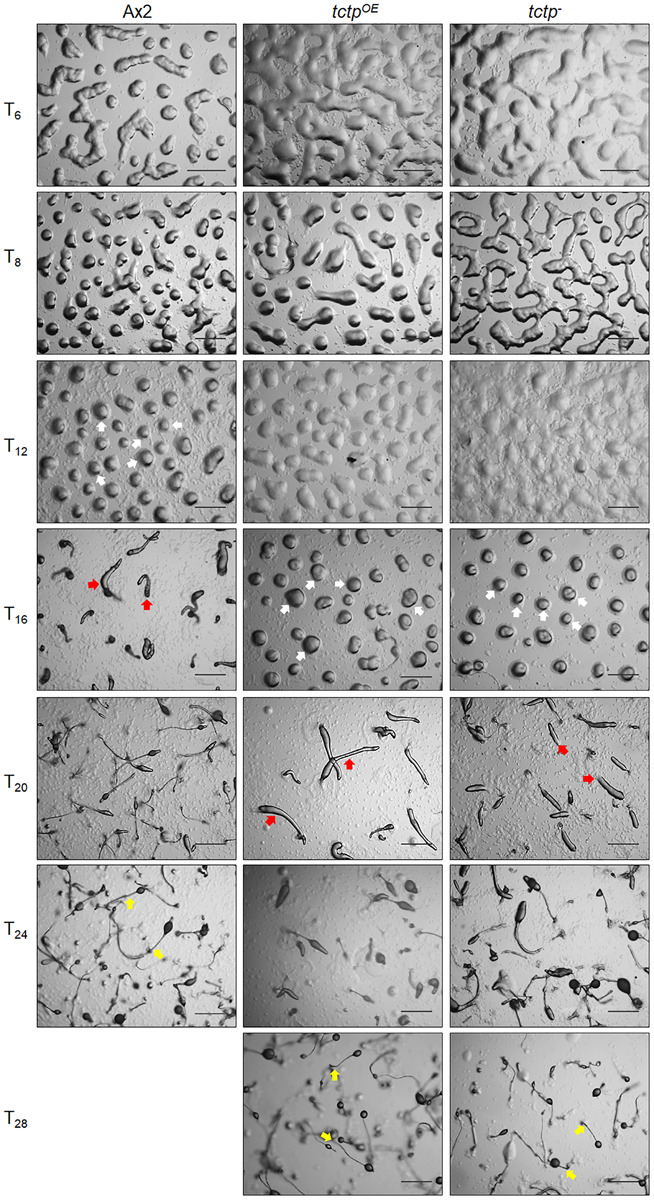
Overexpression/deletion of *tctp* impaired development of *D. discoideum*. Comparative developmental profiles of Ax2, *tctp*^*OE*^, and *tctp*^–^ strains developed at equal cell density and incubated at 22°C for development. White, red, and yellow arrows denoted mound, slug and fruiting body observed in Ax2, *tctp*^*OE*^, and *tctp*^–^ strains, respectively (Scale bar- 200 μm).

**FIGURE 5:**
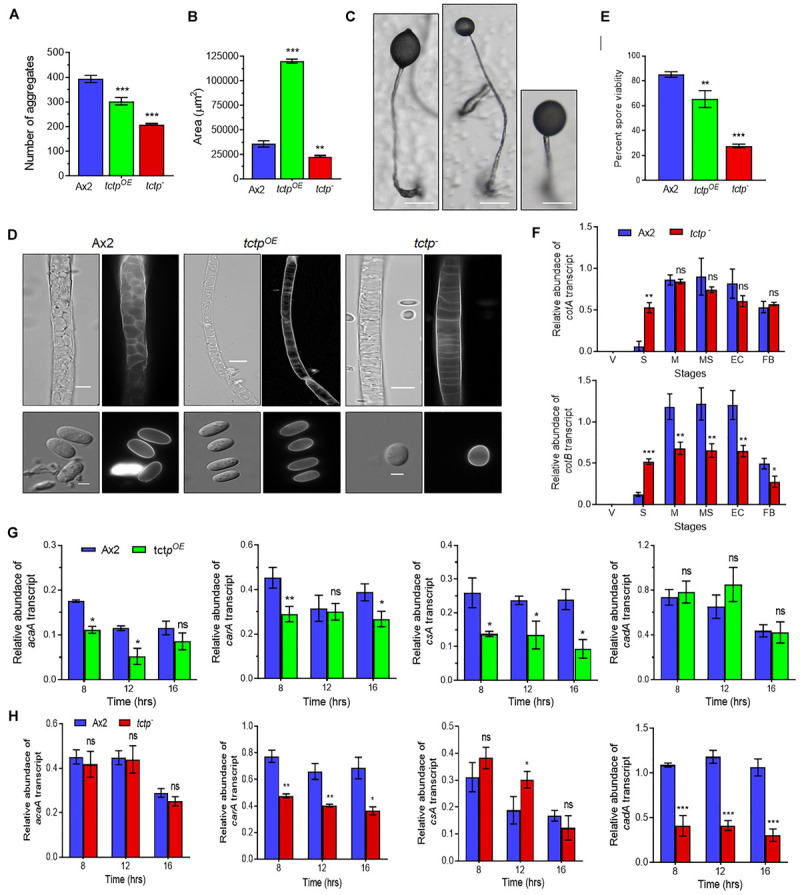
TCTP regulates size of aggregates and fruiting bodies. **(A)** Numbers and **(B)** size of aggregates formed during development of Ax2, *tctp*^*OE*^, and *tctp*^–^ strains. **(C)** Image showing a single fruiting body (*n* = 5; Scale bar = 50 μm). **(D)** Stalk and spore anatomy of Ax2, *tctp*^*OE*^, and *tctp*^–^ strains. **(E)** Percent spore viability showing less viable spores of *tctp*^*OE*^ and *tctp*^–^ strains as compared to Ax2. **(F)** Relative transcripts levels of spore coat A (*cotA*) and B (*cotB*) genes in Ax2 and *tctp*^–^. **(G)** Relative transcripts levels of *acaA*, *carA*, *csA*, and *cadA* early developmentally regulated genes in Ax2 and *tctp*^*OE*^ and **(H)** Ax2 and *tctp*^–^ (The values represent mean ± standard deviation; *n* = 3; ***p < 0.001, **p < 0.01, *p < 0.05; Student’s *t*-test; Scale bar for stalk = 5 μm; Scale bar for spore- 2.5 μm; V- Vegetative, Str- Streaming, M- Mound, MS- Migratory Slug, EC- Early Culminant, and FB- Fruiting body).

To delineate the role of TCTP in multicellular developmental program of *Dictyostelium*, wild type, *tctp*^*OE*^, and *tctp*^–^ cells were plated at a density of 5 × 10^7^ cells/mL on non-nutrient agar and allowed to develop at 22°C following synchronization (Figure 4). Ax2 cells completed its developmental life cycle by 24 h forming aggregation streams by 6 h, aggregates (mounds) by 12 h, slugs by 16 h, and fruiting bodies by 24 h whereas the formation of aggregates was delayed by 4 h in *tctp*^*OE*^ and *tctp*^–^ strains. The *tctp*^*OE*^ and *tctp*^–^ cells formed mound at 16 h, slugs at 20 h and fruiting body at 28 h (Figure 4). Additionally, the number of aggregates were significantly reduced in *tctp*^*OE*^ and *tctp*^–^ strains as compared to Ax2 (Figures 5A,B) but the size of aggregates was 3-fold more in case of *tctp*^*OE*^ whereas *tctp*^–^ showed comparatively smaller aggregates than Ax2. *tctp*^*OE*^ cells formed long and slender slugs as compared to wild type whereas *tctp*^–^ slugs was similar to wild type slugs. Then, we analyzed the fruiting body morphology of wild type, *tctp*^*OE*^ and *tctp*^–^ strains and found that *tctp*^*OE*^ cells formed fruiting body with long, thin stalk and small sorus while *tctp*^–^ cells developed into fruiting body with a small and thick stalk and a large sorus as compared to wild type fruiting bodies (Figure 5C). Moreover, in case of *tctp*^*OE*^ and *tctp*^–^ fruiting bodies stalk cells are arranged in a septate pattern as compared to the polyhedral manner in wild type cells (Figure 5D). Further, cellulose deposition in fruiting bodies of these strains were checked by calcofluor staining and found less cellulose deposition in the septum of *tctp*^–^ stalk (Figure 5D and Supplementary Figure S3A). The spores formed by *tctp*^*OE*^ cells were elliptical similar to Ax2 but *tctp*^–^ spores were round in shape (Figure 5D and Supplementary Figure S3B). *cotA* (spore **CO**a**T** protein, SP96) and *cotB* (spore **CO**a**T** protein, SP70) codes for major spore coat proteins, SP96 and SP70, respectively, that are necessary for the integrity of the spore coat (Srinivasan et al., 2000). Altered levels of any of these coat proteins are responsible for defect in shape and size of spores formed. Since the shape of *tctp*^–^ spore was largely altered, thus we checked the expression of spore coat genes *cotA* and *cotB* at the mRNA level by semi-quantitative RT-PCR in different stages of development (Figure 5F). We observed the expression of *cotA* remained unaffected, while the transcript of *cotB* was down regulated in *tctp*^–^ strain as compared to wild type (Figure 5F). Collectively, the above results suggest that TCTP is required for timely development and appropriate formation of aggregates and fruiting bodies.

Aberrant fruiting body formation by mutant cells led us to ask whether spores formed by mutant cells were viable or not therefore, spore viability assay was performed. Wild type and *tctp*^*OE*^ cells produced spores with average spore viability of 85.1 ± 1.7 and 65.3 ± 5.5%, respectively, whereas *tctp*^–^ cells produced 27.7 ± 1.2% viable spores (Figure 5E). Therefore, TCTP is essential for maintaining the viability of *Dictyostelium* spores.

### mRNA Expression Analysis of Early Developmentally Regulated Genes

Initial development of *Dictyostelium* is mainly regulated by proteins involved in aggregation such as *acaA (adenyle cyclase aggregate A), carA* (*cAMP receptor A*), cell adhesion molecules *csA (contact site A)*, and *cadA (Calcium-dependent ADhesion). acaA* and *carA* are major components for cAMP signal relay. Since, we observed delay in initiation of streaming we checked the mRNA expression of *acaA* and *carA* in Ax2, *tctp*^*OE*^, and *tctp*^–^ strains (Figures 5G,H). The transcript level of *acaA* remain unaffected while *tctp*^*OE*^ showed reduced expression at 8 h (Figure 5G). The mRNA expression of *carA* was significantly down regulated in *tctp*^*OE*^ at 8 h and *tctp*^–^ at 8, 12, and 16 h. Furthermore, we checked the mRNA expression of cell adhesion molecules *csA* and *cadA* that are required for aggregate formation in early development. *cadA* expression remained unchanged and *csA* level was significantly reduced in *tctp*^*OE*^ cells resulting in larger aggregate formation. The *cadA* mRNA was down regulated and *csA* expression was up regulated at 12 h contributing to decrease cell-cell adhesion and stream break up in *tctp*^–^ aggregates (Figure 5H). The above data suggest that reduced *acaA* and/or *carA* may be responsible for the delay in development and altered *cadA* and/or *csA* expression contribute to altered aggregates formation in *tctp*^*OE*^ and *tctp*^–^ strains.

### Disruption of *tctp* Alters Spatial Cell Type Patterning

**FIGURE 6:**
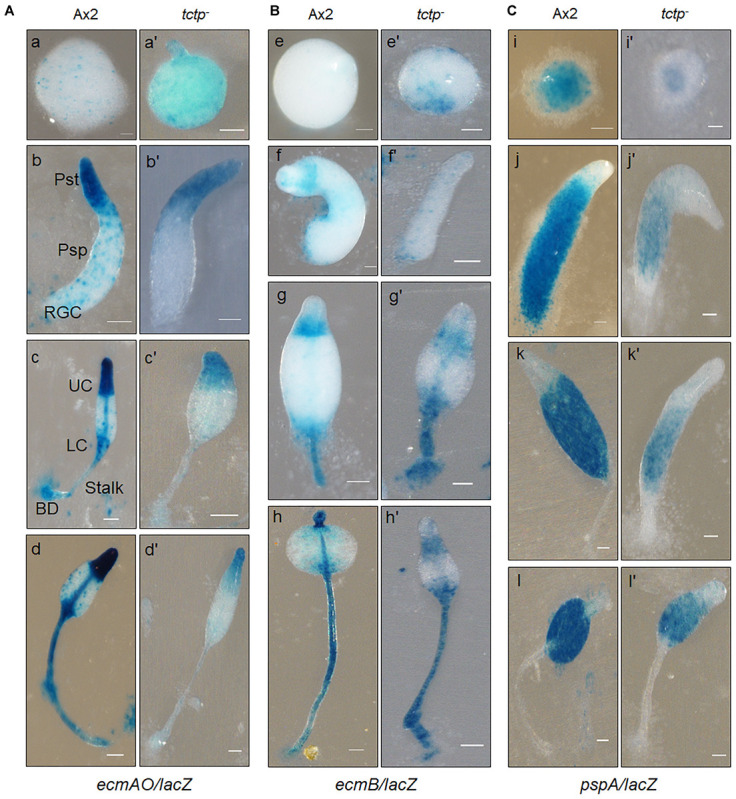
Spatial distribution of prestalk/prespore cells using cell type specific marker genes in Ax2 and *tctp*^–^ cells. Wild type and *tctp*^–^ cells expressing *lacZ* under the prestalk specific promoter **(A)** *ecmAO* [*ecmAO/lacZ*], **(B)** *ecmB* [*ecmB*/*lacZ*], and **(C)** prespore (*pspA or d19*) specific promoters [*pspA/lacZ*]. Various developmental structures were collected, fixed and histochemical staining was performed with X-gal. [Ax2 (A–T) and *tctp*^–^ (A’–T’) developmental structures; Pst, prestalk; Psp, prespore; UC, upper cup; LC, lower cup; BD, basal disk; RGC, rear-guard cells; and Scale bar- 50 μm].

Aberrant fruiting body formation by mutant cells incited us to study its role in cell type differentiation and patterning. The spatial distribution of prespore/prestalk cells in multicellular structures developed by wild type and *tctp*^–^ cells was analyzed using *lacZ* reporter fused to promoter of prestalk/prespore specific marker genes such as *ecmAO*, *ecmB, and pspA (d19).* The *ecmAO/lacZ* staining in wild type slugs was observed in the anterior region of slugs and in ALCs (anterior-like cells) of posterior region while in *tctp*^–^ mound it was found in anterior region but absent in ALCs of posterior region of slug. In *tctp*^–^ early culminant and culminant, *ecmAO/lacZ* staining was absent in the lower cup, basal disk, and the stalk tube (Figure 6A). In wild type, the *ecmB/lacZ* staining was observed in the central core region of prestalk cells of migrating slugs and distributed in the basal disk, upper and lower cups of fruiting bodies formed (Figure 6B). In *tctp*^–^ cells, *ecmB/lacZ* expression is precocious as found at mound stage when compared with the wild type. The *ecmB/lacZ* staining in *tctp*^–^ early and late culminant was found similar to wild type. The *pspA/lacZ* staining was found in the prespore/spore region of the multicellular structures developed but the *pspA/lacZ* staining region was reduced in *tctp*^–^ slugs, early and late culminant (Figure 6C). Thus, we suggest that TCTP plays an important role in the distribution of cell types in multicellular structures.

### *tctp*^–^ Cells Exhibit More Tendency Toward Prestalk/Stalk Region

**FIGURE 7:**
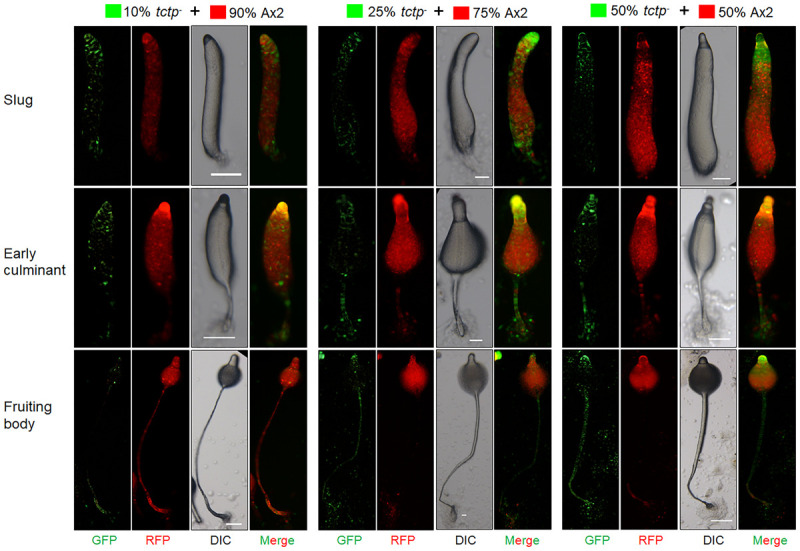
*tctp*^–^ cells exclusively occupied prestalk/stalk region in chimeras. RFP-tagged Ax2 cells and GFP tagged *tctp*^–^ cells were mixed in varying proportions (10, 25, and 50% GFP marked *tctp*^–^ cells and 90, 75, and 50% RFP marked Ax2 cells) were allowed to co-develop on non-nutrient agar until the formation of migratory slug, early and late culminant. Both, DIC and fluorescence images during development were captured using Nikon SMZ1500 fluorescence microscope. (Scale bar- 100 μm; *n* = 4).

**FIGURE 8:**
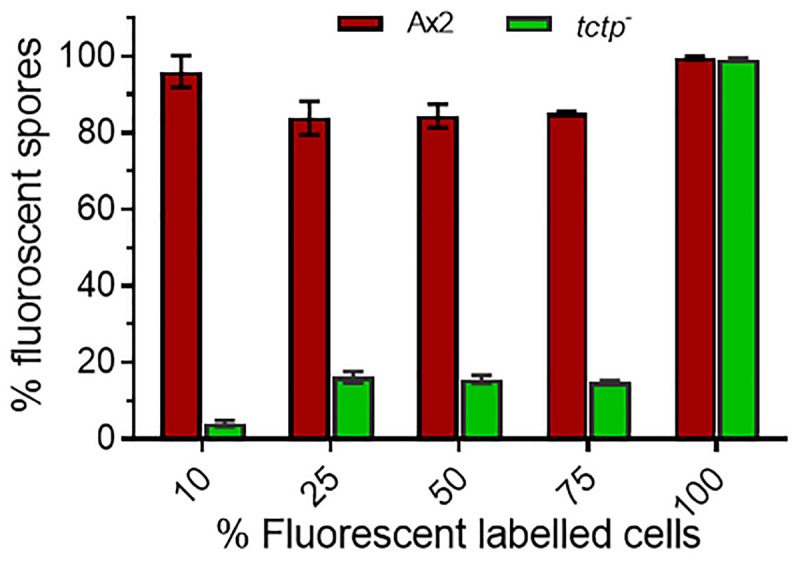
Percentage (%) spore count of Ax2 and *tctp*^–^ cells in chimeric fruiting bodies. GFP tagged *tctp*^–^ cells were mixed in various proportion as given above with RFP-tagged wild type cells and cell suspensions were allowed to co-develop. About 100–200 spores per fruiting body and 10–15 fruiting bodies were counted and scored.

To trace the fate of *tctp*^–^ cells in a chimera, cell types mixing experiments were performed in which Ax2 (RFP-tagged) and *tctp*^–^ (GFP-tagged) mixed in varying ratios of 9:1, 3:1, 1:1, and 1:3 and allowed to co-develop (Figure 7 and Supplementary Figures S4–S6). Chimeras are the mixture of cells from two or more genetically different backgrounds. The differential presence of wild type and/or mutant cells in development and differentiation can be clearly observed by chimera study. Here, we wanted to observe the differential preference of mutant cells in presence of wild type cells. During early development, Ax2 and *tctp*^–^ cells contribute equally in formation of aggregation centers of various chimeras (1:9, 1:3, 1:1, and 3:1 of *tctp*^–^ and Ax2 cells; Supplementary Figure S4). As the development proceed further, in chimera of 10% *tctp*^–^ and 90% wild type cells, we observed that *tctp*^–^ cells occupied the prestalk/stalk region while the Ax2 cells occupied the prespore/spore region. During slug formation, *tctp*^–^ cells majorly occupied the anterior prestalk region, rear-guard and ALCs (Figure 7 and Supplementary Figure S5). At early culminant stage, *tctp*^–^ cells occupied the anterior prestalk region, ALCs, and basal disk. At culminant, it majorly contributed to anterior tip region, upper and lower stalk portion and basal disk (Figure 7 and Supplementary Figure S5). This trend was observed as the percentage of *tctp*^–^ cells increased further as it also occupied entire stalk and basal disk region. Next, the contribution of *tctp*^–^ cells in spore formation was assessed and it exhibited decreased contribution in the prespore/spore region as compared to wild type cells. The contribution of *tctp*^–^ cells in chimeras of varying proportion, i.e., 10, 25, 50, 75, and 90% of *tctp*^–^ cells with Ax2 cells resulted in chimeric spore formation in which *tctp*^–^ cells contributed only to 1.95 ± 0.35, 11 ± 1, 15 ± 1, 15.5 ± 0.5, and 49 ± 9% (Figure 8). Our results suggested that in chimeras with wild type cells, *tctp*^–^ cells differentiated into prestalk/stalk cells preferentially, further suggesting that TCTP is a crucial component controlling the equilibrium between prestalk and prespore cell-types.

## Discussion

Translationally controlled tumor protein is ubiquitously expressed, highly conserved protein present in various type of cells and tissues implicating its essential role in normal cells. This conservation is also observed in the protozoans, such as *Plasmodium*, *Toxoplasma*, and *Dictyostelium* (Bhisutthibhan et al., 1998; Zheng et al., 2018). It is involved in regulation of cell cycle, development, cytoskeleton, autophagy, and apoptosis. It plays a vital role in the growth and development of an organism by regulating the main physiological events. However, the biologic function of this protein in social amoeba such as *Dictyostelium* has never been investigated. Sequence analysis suggested that the key residues are conserved throughout the evolution. Previous reports demonstrated that Glutamate12 (Glu12) present in the loop region of *Drosophila* TCTP is an important residue for Rheb (Ras homologue enrichment in brain) function that activates TOR (Target of rapamycin) but it is contradictory that TCTP has role in mTORC1 signaling pathway (Chen et al., 2007; Hsu et al., 2007; Rehmann et al., 2008). Conservation of Glu12 in *Dd*TCTP thus open the possibility of Rheb function in lower eukaryotes. Phylogenetic studies suggested that *Dd*TCTP is highly conserved protein in eukaryotes. Residues important for microtubules and Ca^2+^ binding are also conserved in different species studied (Bommer and Thiele, 2004; Graidist et al., 2007). In contrary, Ser46 and Ser64 residues which phosphorylated by the Plk are not conserved in lower eukaryotes and found only in mammalian TCTP (Yarm, 2002). Higher expression of *Dd*TCTP mRNA was observed in vegetative or initial stages, suggested that *Dd*TCTP may have a role in cell proliferation and cell division. As the complete knockout of *tctp* gene was lethal it limited the understanding of its functions. Here, we have studied the functions of TCTP during growth and development of *D. discoideum* with the help of making overexpressor and knockout strains. Overexpression and disruption of *tctp* in *Dictyostelium* displayed reduced cell proliferation. Previous studies also suggested that overexpression of TCTP resulted in inhibition of cell proliferation in cell lines (Gachet et al., 1999). It has tubulin binding properties and found in association with tubulin or microtubules in cell cycle phases, its overexpression causes rearrangement of microtubules and stability. Disruption of *tctp* exhibited increased cell-size, which could be due to multi-nucleation. The size of organs or organism depends on the number and size of the cells that constitute them. Overexpression and disruption of *tctp* resulted in delayed aggregation stream formation and decelerated *acaA* and *carA* expression implying that an optimum level of TCTP is essential for normal early gene expression and early development. There are several genes whose timely expression and accurate level is important for regulation of early and late development such as overexpression or disruption of *srsA* exhibited delayed aggregation and reduced expression of *acaA* and *carA* along with aberrant fruiting body formation suggesting its role in the regulation of development (Sasaki et al., 2008). The genes *acaA* (adenylyl cyclase A) and *cAR* (cAMP receptor) require to mediate the cAMP signaling during early development such as aggregation. During aggregation, *Dictyostelium* cells secretes and sense cAMP. ACA (Adenylate cyclase A) is a membrane protein which convert ATP to cAMP and cAMP get transported outside of the cells. Then cAMP bind with surrounding cells through cAR (cAMP receptor). Lacking of *acaA and carA* genes result abolish aggregation and delay in early development (Sun et al., 1990; Pitt et al., 1993). *tctp*^*OE*^ cells form less but large-sized aggregates while *tctp*? cells form less but small-sized aggregates suggesting its involvement in regulation of aggregate size. Previously, various genes of *Dictyostelium* were reported in regulating aggregate size such as *ctnA*, *srsA*, and *ampk* (Okuwa et al., 2001; Sasaki et al., 2008; Maurya et al., 2017). *tctp*^*OE*^ cells form long slugs and fruiting bodies with long stalks and small sori that has elliptical spores whereas *tctp*^–^ cells developed into fruiting bodies with small stalks and large sori having round spores as compared to wild type suggesting its role in terminal differentiation. The round spores formed by *tctp*^–^ fruiting bodies could be due to decreased expression of spore coat protein, *cotB* that has been previously known to maintain integrity of spore. Overexpression of *tctp* resulted in long fruiting bodies while deletion of it resulted in small fruiting bodies suggesting the function of TCTP in cell type differentiation and patterning for maintenance and establishment of proper proportioning and patterning. Interestingly, spatial distribution of prespore/prestalk cell type specific markers were aberrant and mis-localized in the multicellular structures formed by *tctp*? cells as compared to Ax2. The aberrant localization of *ecmAO*/lacZ in *tctp*^–^ alluding its role in cell type patterning. Our results showed that TCTP is involved in cell type patterning and regulates the ratio of prestalk/prespore cells in multicellular structures formed. A number of genes have been shown to play a role in cell type proportioning and spatial patterning in *Dictyostelium*. In *Mekk*α null cells, there was a two-fold increase in pstO compartment and concomitant decrease in prespore region (Chung et al., 1998). Disruption of homeobox containing gene, *warai* led to doubling of pstO compartment (Han and Firtel, 1998). Overexpression of rZIP led to reduce prespore and enhanced prestalk expression (Balint-Kurti et al., 1997; Ginsburg and Kimmel, 1997). Furthermore, cell lineage study with the help of chimeric mixtures of Ax2 and *tctp*^–^ in different proportions showed occupancy of both Ax2 and *tctp*^–^ cells in different regions of multicellular structures developed. Chimeras are mixture of cells from two or more genetically different backgrounds. Our results clearly show the tendency of *tctp* null cells to form prestalk cells in presence of wild type. It occupied the prestalk regions like rear-guard cells, the anterior-like cells, lower cup, basal disk, and stalk regions. When the percentage of the null cells was increased in the chimeras, *tctp*? cells could contribute to spore cells though the spore forming tendency by the null cells was very low. The spore count from various chimeric mixtures substantiated our results that *tctp*? cells has less proclivity in spore formation in presence of wild type cells. The deletion of *tctp* allowed the cells to occupy the dying cell population suggesting that it may be required to regulate the spore differentiation. Thus, TCTP may contribute toward both spore and stalk cell differentiation but the question arises, how TCTP regulate spore/stalk differentiation in this organism? Whether TCTP is directly involved in regulating differentiation and patterning or it regulates expression of other genes involved in this process so, further studies are needed to be explored to clarify its exact role in cell type differentiation and patterning.

## Conclusion

Our study suggests that TCTP regulates the cell size and cell proliferation in *Dictyostelium*. It is required for timely development and correct aggregation formation. Further, it is involved in maintaining prespore/prestalk or spore/stalk ratio and cell type differentiation and patterning.

## Data Availability Statement

All datasets presented in this study are included in the article/Supplementary Material.

## Author Contributions

RK, RM, and SS conceived and designed the study, analyzed the data, and wrote the manuscript. RK and RM performed the experiments. All authors read and approved the final version of the manuscript.

## Conflict of Interest

The authors declare that the research was conducted in the absence of any commercial or financial relationships that could be construed as a potential conflict of interest.
