# Supplementary material for: Investigating the Role of Translationally Control Tumor Protein in Growth, Development and Differentiation of Dictyostelium discoideum
Source: Front Cell Dev Biol. 2020 Aug 7;8:742. doi: 10.3389/fcell.2020.00742 (PMC7426469; doi:10.3389/fcell.2020.00742)
Supplement: Supplementary file 1 [file Data_Sheet_1.PDF]

# Supplementary material

## Investigating the role of translationally control tumour protein in growth, development and differentiation of *Dictyostelium discoideum*

Rakesh Kumar[#], Ranjana Maurya[#] and Shweta Saran*

Cell and Developmental Biology Lab, School of Life Sciences, Jawaharlal Nehru University, New Delhi-110067

***Corresponding author**: Shweta Saran

Cell and Developmental Biology Lab, School of Life Sciences, Jawaharlal Nehru University, New Delhi, India-110067

[#]Both the authors contributed equally

**Email:**

RK: rakeshkapoor.jnu@gmail.com

RM: ranjanamauryasls@gmail.com

SS: ssaran@mail.jnu.ac.in; shweta_saran@hotmail.com

**Running title:** Role of TCTP in growth and development of *Dictyostelium*

Supplementary figures: 6

Supplementary tables: 2

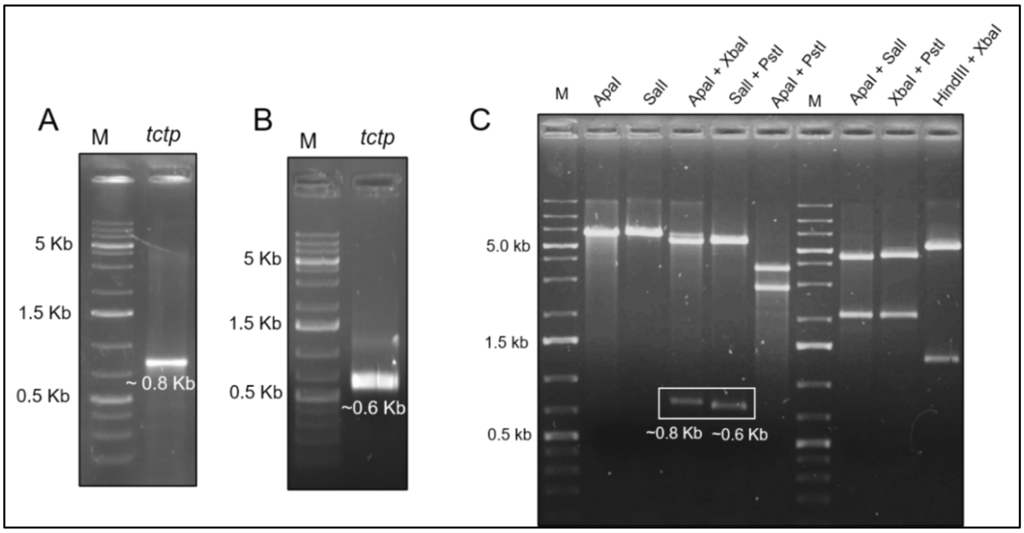


**Figure S1.** ***tctp* knockout strain preparation and validation.** (A) PCR amplification of 5' targeting region have amplicon size ~0.8kb, (B) PCR amplification of 3' targeting region have amplicon size ~0.6kb, (C) Confirmation of 5' and 3' targeting region and BSR (Blasticidin Resistance) cassette via restriction digestions and (D) Gel picture displayed the confirmation of *tctp* overexpressor and knockout strains by RT-PCR from cDNA of Ax2, *tctp*$^{OE}$ and *tctp*$^{-}$. [M is DNA marker; Kb - kilo basepair; Target fragments were highlighted in rectangular box in right panel].

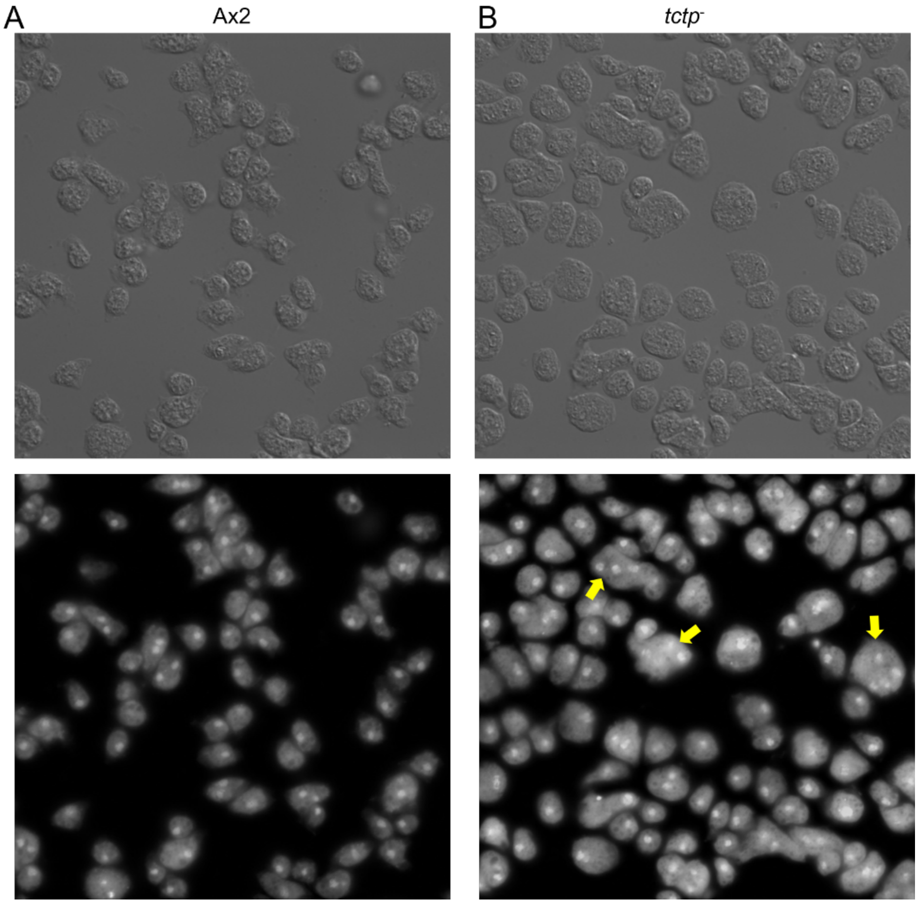


**Figure S2.** **Cell size analysis through microscopy.** (A) Microscopic images of Ax2 brightfield (upper) and DAPI (lower). (B) Microscopic images of *tctp*$^{-}$ brightfield (upper) and DAPI (lower). Multinucleated cells are highlighted by yellow arrows.

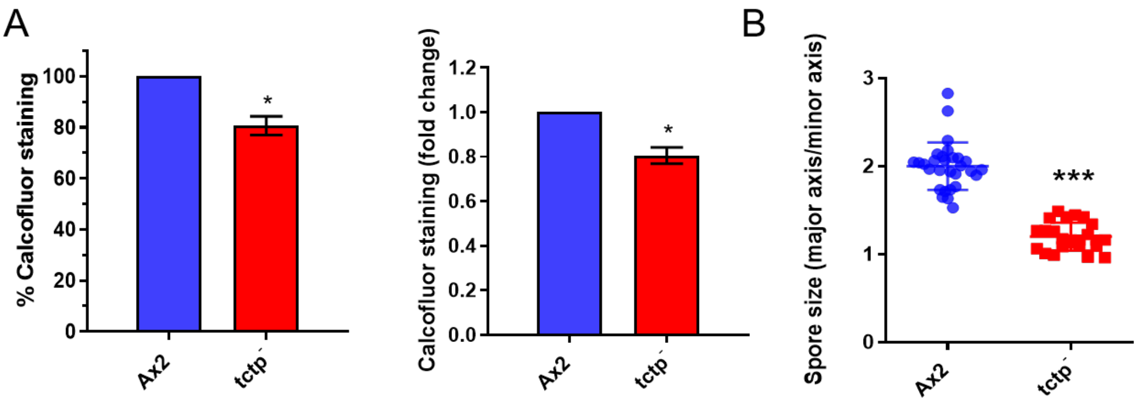


**Figure S3. Quantification of calcofluor staining in stalk and measurement of spore size of Ax2 and *tctp*⁻ strains. (A)** Percent calcofluor staining (B) Spore size analysis [The values represent mean ± standard error mean; n=3; ***p <0.001, *p < 0.05; Student's t-test].

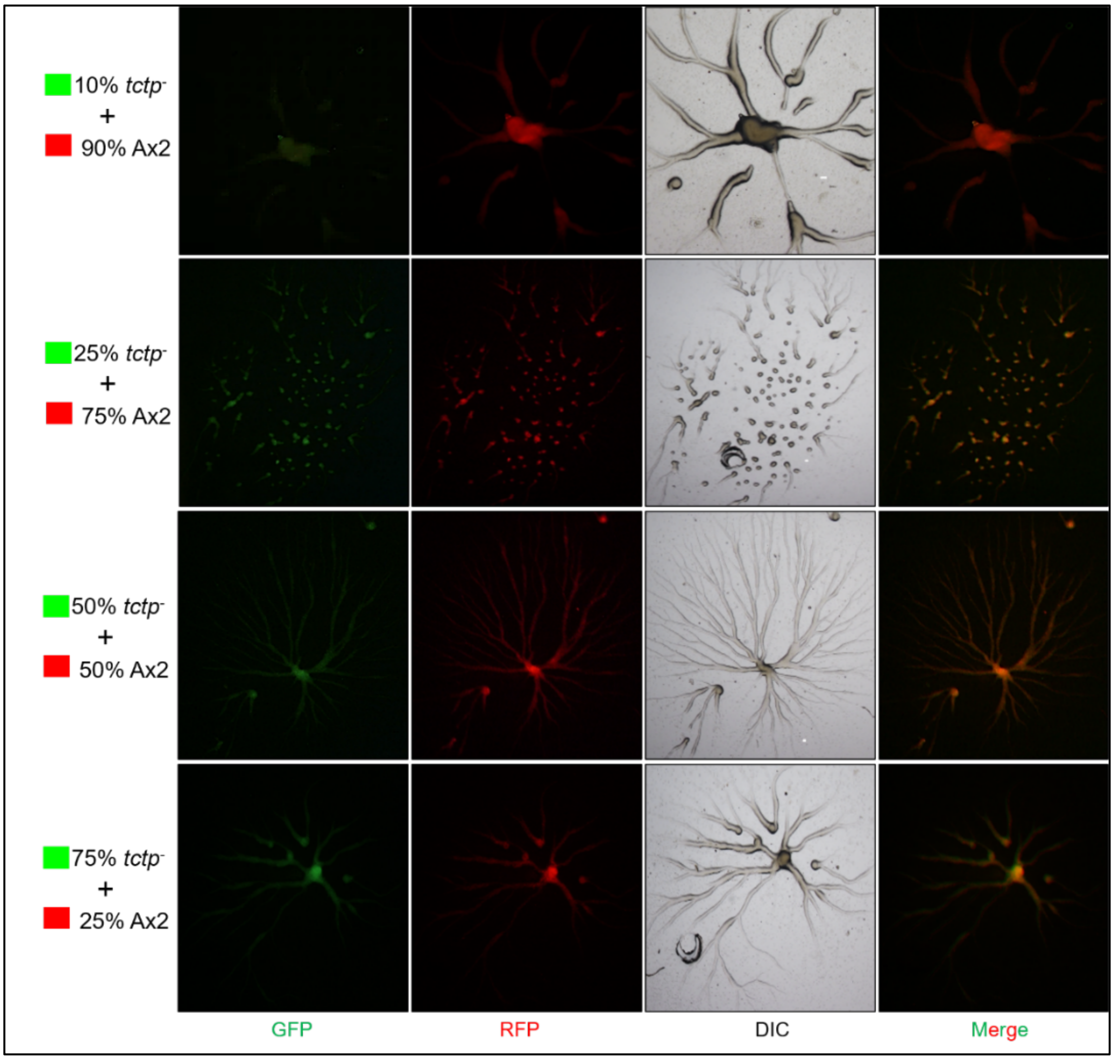


**Figure S4. Streaming during the chimeras of Ax2 and *tctp*⁻ cells developed in different ratios.** Multicellular structures of *Dictyostelium* represented by Ax2 as red and *tctp*⁻ as green. [GFP- Green fluorescent protein; RFP- Red fluorescent protein; DIC- Differential interference contrast; Scale bar- 100µm].

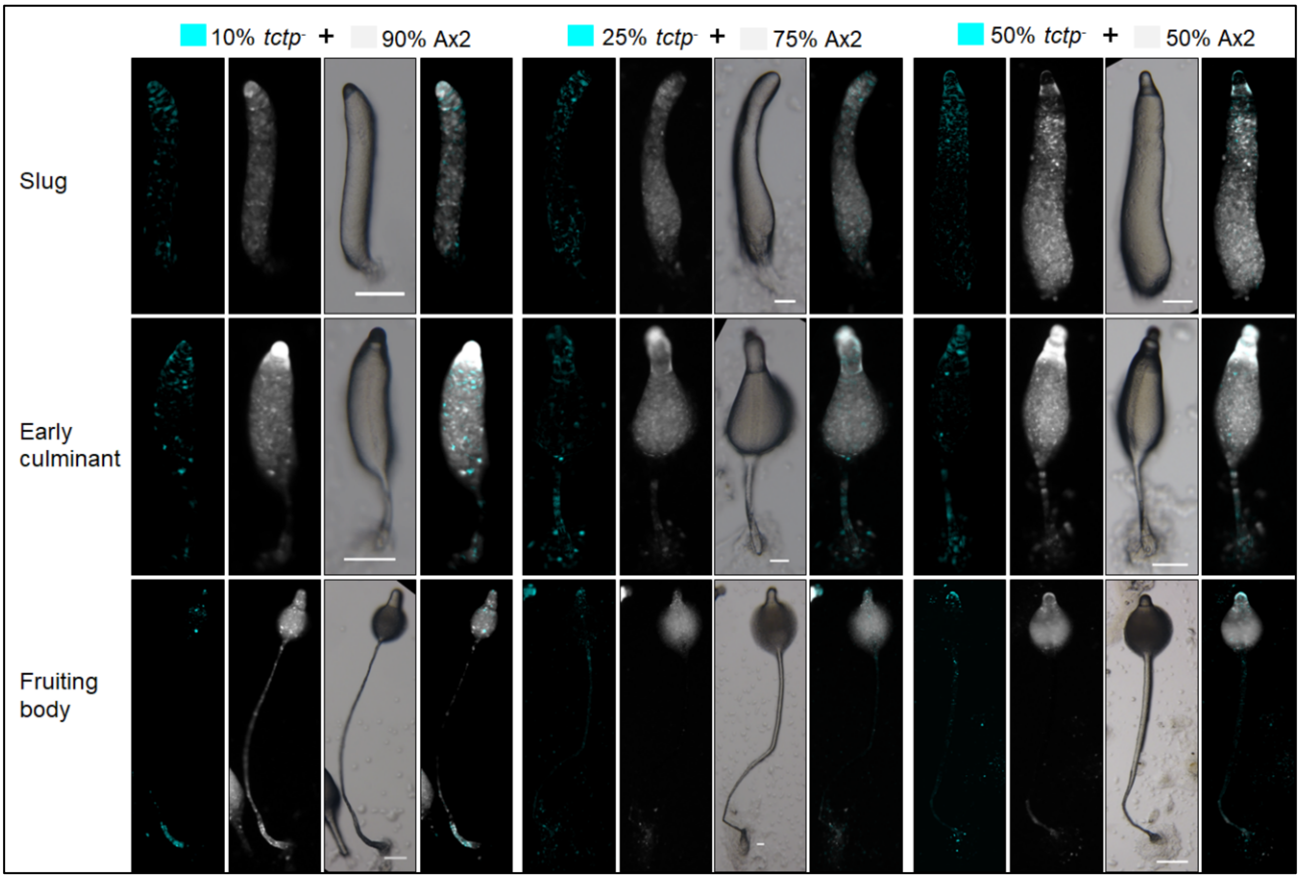


**Figure S5.** ***tctp*⁻ cells exclusively occupied prestalk/stalk region in chimeras.** RFP-tagged Ax2 cells are shown in cyan and GFP tagged *tctp*⁻ cells are shown in greyscale.

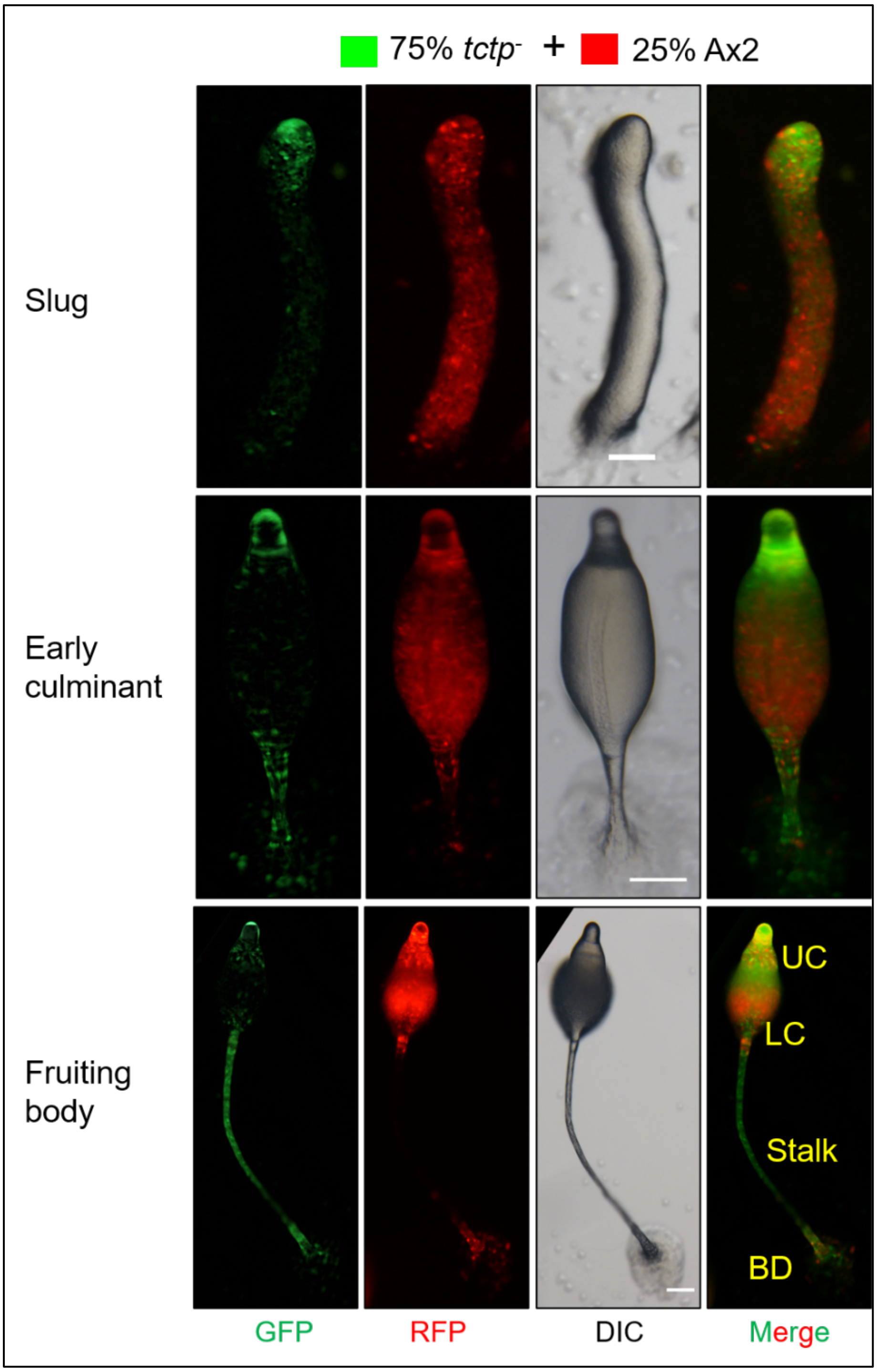


**Figure S6. Chimeras of Ax2 and *tctp*⁻ cells developed in 3:1 ratio.** Multicellular structures of *Dictyostelium* represented by Ax2 as red and *tctp*⁻ as green. [GFP- Green fluorescent protein; RFP- Red fluorescent protein; DIC- Differential interference contrast; UC- upper cup; LC- lower cup; BD- basal disc; Scale bar- 100µm].

**Table S1. List of oligonucleotides used for *tctp⁻* knockout screening**

| Gene name (gene id) | Primer number | Primer 5'-------------3' |
|---|---|---|
| *tctp* (DDB_G0282853) | P1 | CGATGGGCCCTCAACTCTGGTATTGTGGAATCAC |
| | P2 | TGCAGTTTCGAATGGACAAA |
| | P3 | TTTGTCCATTCGAAACTGCA |
| | P4 | GTGACTGCAGAACCAGAACCAGTTATTGAAG |
| | P5 | ATGTACAATGCTAAAGTTTCCTTC |
| | P6 | ATGAGTTTGGTCTCCTTAAAATTG |

**Table S2. List of oligonucleotides used in the study**

| Gene name (gene id) | Primer | Primer 5'-------------3' | Genomic position | Amplicon size (bp) |
|---|---|---|---|---|
| *tctp*-RT<br>(DDB_G0282853) | FP<br>RP | ATGCCTACCCAATGACTG<br>TTAACTGATTCTAAACCG | 1334-1351<br>1903-1886 | 468 |
| *rnlA*-RT<br>(DDB_G0294034) | FP<br>RP | TGAATTGAAGTCTGAGTAAACGG<br>TAGATAGGGACCAAACTGTCTCAC | 1795-1817<br>3065-3042 | 721 |
| *tctp*$^{OE}$<br>(DDB_G0282853) | FP<br>RP | ACGCGGATCCAGAGTTTTCAAAGATATT<br>ACGGCTCGAGAATATTTAACTGATTCTAA | 1004-1023<br>1907-1890 | 921 |
| *tctp⁻* 5' targeting region | FP<br>RP | CGATGGGCCCTCAACTCTGGTATTGTGGAA TCAC<br>GACTTCTAGAGTTGATTTCAGTCATT GGGTAGGC | 513-536<br>1359-1336 | 847 |
| *tctp⁻* 3' targeting region | FP<br>RP | CTCAGTCGACGTGAAAATATGGATGCTGATGGTC<br>GTGACTGCAGAACCAGAACCAGTTATTGAAG | 1697-1720<br>2493-2473 | 797 |
| *acaA*-RT<br>(DDB_G0281545) | FP<br>RT | AGTACACCACATAATAATAATCAT<br>CTCTGGAATTACAATATCTCTCTT | 1304-1327<br>2503-2480 | 1076 |
| *carA*-RT<br>(DDB_G0273397) | FP<br>RP | TGTATGGCAGTGTTGATTGGT<br>ATGGTGATGGATTGTTATTGT | 1082-1102<br>2044-2024 | 819 |
| *csA*-RT<br>(DDB_G0289073) | FP<br>RP | GTGAACGACTCTATTAACTCTGCT<br>AGTTGGAGTGTCTGGAATTGTATA | 406-430<br>1374-1350 | 968 |
| *cadA*-RT<br>(DBB_G0285793) | FP<br>RP | TCTGTTGATGCAAATAAAGTAAAA<br>ATAGTCATATGGTGTATGTGTTTG | 4-28<br>583-559 | 468 |
| *cotA*-RT<br>(DDB_G0276941) | FP<br>RP | TAATAAGCTTGAAAGATAATTGTGGAGAAGGTGGTGATG<br>TTATCTCGAGGGAAGAGCTTGATGATGCAGATGAAG | 1385-1414<br>2713-2686 | 1300 |
| *cotB*-RT<br>(DDB_G0276761) | FP<br>RP | GGTCAAGCTTAGAGATAGTAACGATTGTCTTGCTAG<br>TTACCTCGAGATAGTTGATGGATTGATACAGATTGG | 1468-1493<br>2579-2554 | 1112 |
